# Photomotility of polymers

**DOI:** 10.1038/ncomms13260

**Published:** 2016-11-10

**Authors:** Jeong Jae Wie, M. Ravi Shankar, Timothy J. White

**Affiliations:** 1 Air Force Research Laboratory, Materials & Manufacturing Directorate, Wright-Patterson Air Force Base, Ohio 45433-7750, USA; 2 Azimuth Corporation, Dayton, Ohio 45432, USA; 3 Inha University, Department of Polymer Science and Engineering, Incheon 22212, South Korea; 4 University of Pittsburgh, Department of Industrial Engineering, Pittsburgh, Pennsylvania 15261, USA

## Abstract

Light is distinguished as a contactless energy source for microscale devices as it can be directed from remote distances, rapidly turned on or off, spatially modulated across length scales, polarized, or varied in intensity. Motivated in part by these nascent properties of light, transducing photonic stimuli into macroscopic deformation of materials systems has been examined in the last half-century. Here we report photoinduced motion (photomotility) in monolithic polymer films prepared from azobenzene-functionalized liquid crystalline polymer networks (azo-LCNs). Leveraging the twisted-nematic orientation, irradiation with broad spectrum ultraviolet–visible light (320–500 nm) transforms the films from flat sheets to spiral ribbons, which subsequently translate large distances with continuous irradiation on an arbitrary surface. The motion results from a complex interplay of photochemistry and mechanics. We demonstrate directional control, as well as climbing.

## Abstract (web summary)

The demand for soft robots urges the development of new light-responsive materials for remotely powered actuation. Here, Wie *et al*. show directional motion over centimeter scales using azobenzene-functionalized liquid crystalline polymer films upon continuous radiation from ultraviolet to visible light.

## Graphical abstract

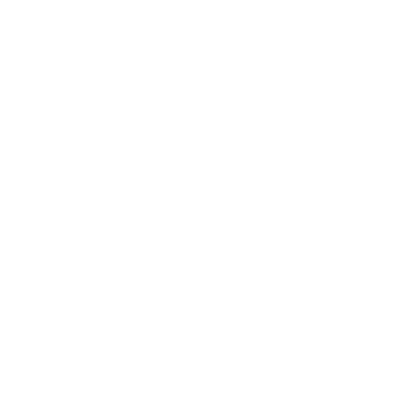

As with the organisms that inspire the mechanical design, soft robots and the subsystems that compose them must efficiently source and transduce energy into sufficient impulses to exceed the threshold power-to-weight ratio for translation. In miniaturized systems, the weight penalty of power sources can be prohibitive. Accordingly, remotely and wirelessly powered actuation is appealing for achieving sustained locomotion in miniaturized systems. One recent study reports gait induced with temporally modulated magnetic fields^1^. Of the potential stimuli capable of remotely powering a system, light is also appealing due to the speed, ease of temporal control and opportunity to spatially localize the mechanical response^2^.

Many recent examinations have examined materials in which the azobenzene chromophore is covalently bonded or doped as a guest into a polymer network^3^,^4^. Photomechanical effects in crystalline materials have also been subject to recent research, including demonstrations of bending, jumping and twisting^5^,^6^. Photomechanical effects in polymers and crystalline materials have been subject to a number of recent reviews^7^,^8^,^9^,^10^,^11^. Although not a requirement for the realization of photoinduced deformation in polymers, considerable recent attention has focused on examinations of azobenzene-functionalized liquid crystalline polymer networks (azo-LCNs) and elastomers (azo-LCEs). One of the primary benefits of liquid crystalline polymer networks and elastomers in mechanical applications is the ability to generate monolithic yet designed materials with local variation in the spatial (in the plane) and hierarchical (through thickness) orientation of the materials^12^. In this way, programming the anisotropy of azo-LCNs could emulate the anisotropic mechanics evident in many of the natural examples of locomotion described hereto.

Here, we demonstrate photoinduced motion (photomotility) of thin strips of a photoresponsive polymer that transform from flat into spiralled structures on irradiation. The monolithic and photoresponsive polymeric material is an azo-LCN composed with 20 wt% of azobenzene crosslinker, identical in composition to those described in prior reports^13^,^14^. The photomotility is a spontaneous mechanical response of these anisotropic materials where the intrinsic granularity of the actuation mechanisms is at the molecular level (*trans-cis* isomerization) and offers refined levels of modularity for tuning mechanical adaptivity. Due to the hierarchical (through thickness) variation in the director profile (twisted nematic orientation) offsetting the alignment of the director to the principal axes of the strips results in the formation of spiralled shapes^15^,^16^,^17^,^18^,^19^. We demonstrate that irradiation of these materials can result in seemingly perpetual photomotility. The directionality of the photomotility is programmed by the orientation of the anisotropy to the principal axes of the specimens. The motion occurs without modulating or multiplexing the actinic light source and on an arbitrary surface. This is distinct from prior engineered constructs that require a temporally modulated stressor in conjunction with anisotropic surface interaction to manifest directional motion^20^,^21^,^22^,^23^,^24^. The material by itself is the motile device without requiring a composite, multimaterial design or other special conditions. By directly transducing photons into motion, the weight penalty of articulated mechanisms, actuators or on-board power sources is eliminated.

## Results

### Photomotility

**Figure 1:**
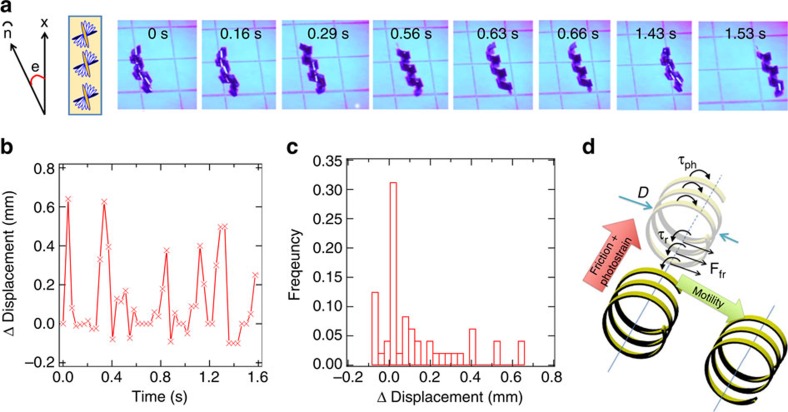
Photomotility of a polymeric strip. (**a**) Light induced motion (photomotility) of a thin strip composed of an azo-LCNs in the twisted nematic geometry aligned with the nematic director offset +15° (top) and −75° (bottom) to the principle axes of the strip. On irradiation with 200 mW cm^−2^ of 320–500 nm light, the 15 μm thick strip forms a spiral ribbon and to continuous irradiation moves to the right. (**b**) The relative displacement taken from frame by frame analysis is recorded as a function of time. (**c**) A histogram of normalized frequency *versus* relative displacement demonstrates the variability in the motion. (**d**) Schematic of the spiral ribbon force balance illustrating the mechanism of the photomotility.

The photomotility of the polymeric material (azo-LCN) is illustrated in Fig. 1a and can be observed in Supplementary Movie 1. When the 15 mm (*L*) × 1.25 mm (*W*) × 15 μm (*T*) polymeric strips (flat) are placed on a paper substrate, light irradiation (200 mW cm^−2^ of broad spectrum 320–500 nm light generated with a Mercury lamp) first generates a spiralled shape^25^,^26^. Continued irradiation of the samples initiates and sustains motion. The azo-LCN sample in Fig. 1 ‘rolls' from left to right across the substrate. The azo-LCN material is prepared in the twisted nematic conformation, in which the nematic director rotates 90° across the sample thickness. Depicted in Fig. 1a, the director at the two surfaces of the film are aligned +15° and −75° to the principal axes of the strip. Due to the inclusion of a chiral additive, R1011, the rotation of the director profile is right-handed across the sample thickness^21^. The broadband emission (320–500 nm) of the Mercury arc lamp subjects the azobenzene chromophores to simultaneous *trans-cis* and *cis-trans* isomerization; forming a photostationary state concentration. Frame by frame image analysis over a period of 1.6 s illustrates the displacement of the film to continuous irradiation (Fig. 1b). The distribution in the relative displacement of the film is plotted in Fig. 1c described as frequency. In this example, the strip can displace as much as 0.64 mm in a single frame (30 ms) and the motion is inherently intermittent.

Due to the strong absorption of light in the range of 300–450 nm, the azo-LCN films are subject to a light intensity gradient that correspondingly yields a strain gradient across the sample thickness. This strain gradient is further enhanced by the employment of the twisted nematic orientation. Offsetting the nematic director to the principle axes of the samples examined here and in prior reports is known to naturally result in the spontaneous formation of spirals^15^,^16^,^17^. The motion evident in Fig. 1 is distinctive from prior reports^1^,^27^,^28^ of photoinduced motion in azo-LCN materials in that low intensity, single source light irradiation is used to transform a flat specimen into a spiral and subsequently powers the motion to continuing irradiation. The mechanics of the motility is illustrated in Fig. 1d. On irradiation, the azo-LCN samples exhibit contractile strains parallel to the nematic director (*ɛ*_||_), in this instance attributable to the loss of nematic order induced by the *trans-cis* isomerization (for example, the *cis* isomer concentration). The magnitude of the strains is proportional to the intensity of light. The reduction in order parameter produces coupled tensile strains perpendicular to the nematic director in a manner that can be considered an optical Poisson-like effect (*ɛ*_⊥_). The spiral structures observed here and in prior reports^15^,^16^,^17^ are manifestations of shear strain gradients through the thickness, scaling in proportion to *ɛ*_||_–*ɛ*_⊥_. In the conditions of the experiments here, the samples are placed on a surface and irradiated from above, which naturally breaks the symmetry of the spiral. These experimental conditions dictate that the upper portion of the azo-LCN film which is in the optical path seeks to adopt a greater twist in comparison to the portion in which light is shadowed. The rate of accumulation of photostrains in the irradiated portion is rapid under the light intensities examined in this work, which is associated with a finite impulse. The generation of this impulse in the spiral geometry with a broken symmetry between the irradiated and the shadowed regions manifests a net twisting moment τ_ph_ as illustrated in Fig. 1d, which we believe is the key to the motility observed here. This configuration is distinct from prior studies where quasistatic deformation is observed in irradiated twisted-nematic, cantilever-like samples with rigid end supports that are allowed to deform and adopt a helical geometry^29^.

In our experiments, the moments from the photostrains increase the twist in the spiral, which seeks to induce slip between the sample and the surface (paper). Friction (F_fr_), which opposes slip, acts tangential to the sample and in doing so creates the unbalanced force to accelerate the centre of mass (Fig. 1d) at an angle that corresponds to the helical angle. Thus, the impulse from the photostrains is transduced into macroscopic motility by friction. The integral role of friction is evident when the films are placed on or within fluids, where the frictionless conditions prevent motility.

However, motion only ensues when the photogenerated moment overcomes the rolling resistance. Rolling resistance arises due to a superposition of hysteresis in the contact, as well as random asperities on the surface, which together can provide a resisting moment (τ_r_). Rolling resistance is commonly observed in cylindrical objects that require a threshold moment to induce rolling on surfaces, even when it occurs without slip at the interface. Though F_fr_ produces a net force to accelerate the centre of mass, it also produces a moment (*D* F_fr_/2, where *D* is the diameter of the spiral) to oppose the rolling. If sufficient moments from the photo-induced impulse can be generated to overcome the opposition from the τ_r_ and *D* F_fr_/2, rolling ensues. When the sample begins to roll, the irradiation profile of the sample shifts, regenerating the forces and sustaining the photomotility from continuous irradiation. The rolling persists as long as the sample is exposed to light without any discernable change in the photomechanical response as a function of time. This is suggestive of a regenerative mechanism wherein the previously occluded part of the spiral is now directly exposed to the maximum light intensity and responds by an increase in photostrain while the photostrains in the now occluded top portion of the azo-LCN are lessened, regenerating the asymmetry necessary to induce torque to overcome the rolling resistance once again.

### Directionality of motion

**Figure 2:**
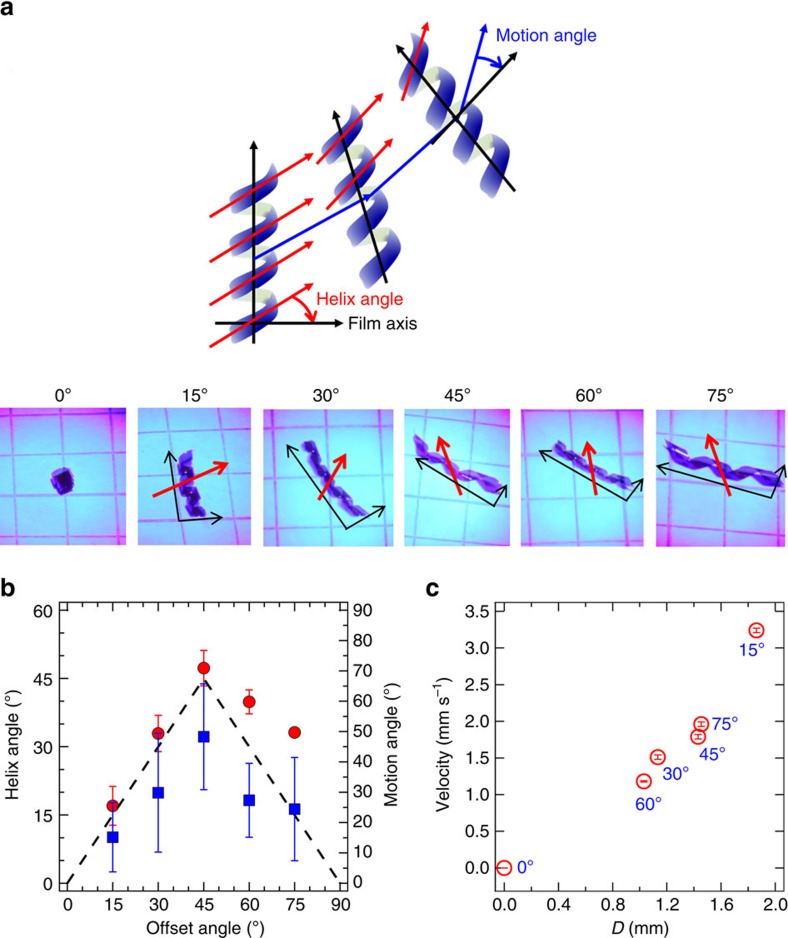
Directional control of photomotility. (**a**) Representative images illustrating the influence of the orientation of the nematic director at the surfaces. Red arrows indicate motile angle of the polymeric coils with respect to the primary axis of coiled film (**b**) The geometry of the helix (Red filled circle, left axis) and the directionality of the locomotion (Blue filled square, right axis) are summarized as a function of offset angle of the top surface of the azo-LCNs to the primary axis of the samples. (**c**) Velocity of the photomotility of the azo-LCNs is plotted against the helix diameter (*D*) of the spiral ribbons. The azo-LCN samples were 15 μm thick. The error bars shown in (**b**) and (**c**) are the standard deviation of each parameter calculated from data measured for 15 seconds monitored at an interval of 0.5 seconds.

The directionality of the displacement is inherent to this mechanism because the photo-induced impulse involves a twist that seeks to create a more tightly coiled spiral. The sign of τ_ph_ (clockwise or counterclockwise) is prebiased by the rotation of the nematic director through the thickness. The twist of the nematic director is controlled by the chiral dopant and the orientation of the nematic director to the principle axes of the samples. Figure 2a and Supplementary Movie 2 visually illustrate the relationship between the orientation of the nematic director, the helical angle and the direction of motion. Specifically, evident in Fig. 2a and Supplementary Movie 2 the principal axes of the strip is varied from 0°/90° to 15°/−75°, 30°/−60°, and 45°/−45°, 60°/−30° and 75°/−15°. As previously reported^16^,^30^, an azo-LCN in the 0°/90° orientation undergoes a large planar deformation coiling back onto itself with no twist along its length and exhibits no photomotility. Unlike the sample in Fig. 1, this condition is dominated by inter–coil interactions and does not build up a moment and accordingly does not exhibit photoinduced motility. However, motion is observable when the director orientation is offset to the principle axes of the strip. The relationship between the film direction, helical angle and the direction of motion is illustrated by the red and blue arrows inset into the images of Fig. 2a, as well explicitly quantified in Fig. 2b. In Fig. 2b, which was taken from the representative movement taken from dozens of experimental examinations, the direction of the motion becomes more arc like in the extremes and is almost linear in the case of the 45°/−45°. As evident in the images, as the helical angle increases the motion angle, which is tangential to the arc-like trajectory, corresponds to a larger angle with respect to the helical axis. Furthermore, the relationship between the direction of the motion and the helical angle the spirals is further emphasized by the symmetry of the relationship evident in Fig. 2b. In this way, Fig. 2b confirms a clear correlation of the helical angle (dictated by the orientation of the nematic director to the surfaces of the azo-LCN) and the direction of motion. Friction that emerges at the surface to oppose the τ_ph_-induced deformation will always be directed along the helical angle. Thus, by affecting the helical angle we are able to direct the arc along which the film moves. In this way, the direction of the motion is preprogrammed into the material itself irrespective of the intensity, direction or collimation of the irradiation source.

Figure 2b and Supplementary Movie 2 examine the relationship between the angle of the motile direction with respect to the axis of the spiral as a function of the offset angle. Note that the offset angle of the strip determines the helical angle of the spiral. This angle is also coincident with the motile direction. This is expected because the direction of F_fr_, which is tangential to the contact to oppose any slip from τ_ph_ will coincide with the helical angle. Since F_fr_ propels the centre of mass, the direction of motion will naturally coincide with the offset angle. While recognizing the motion is inherently intermittent, Fig. 2c illustrates the variation of the average velocity of the samples as a function of the diameter of the helix (*D*). *D* is found to be a function of the offset angle. The correlation between the average velocity and *D* illustrates the influence of helical diameter on motility. This can be rationalized from the variation of rolling resistance as a function of diameter. It has been recognized that larger *D* entails a lower τ_r_ and this will result in greater motility^31^.

### Intensity and photomotility

**Figure 3:**
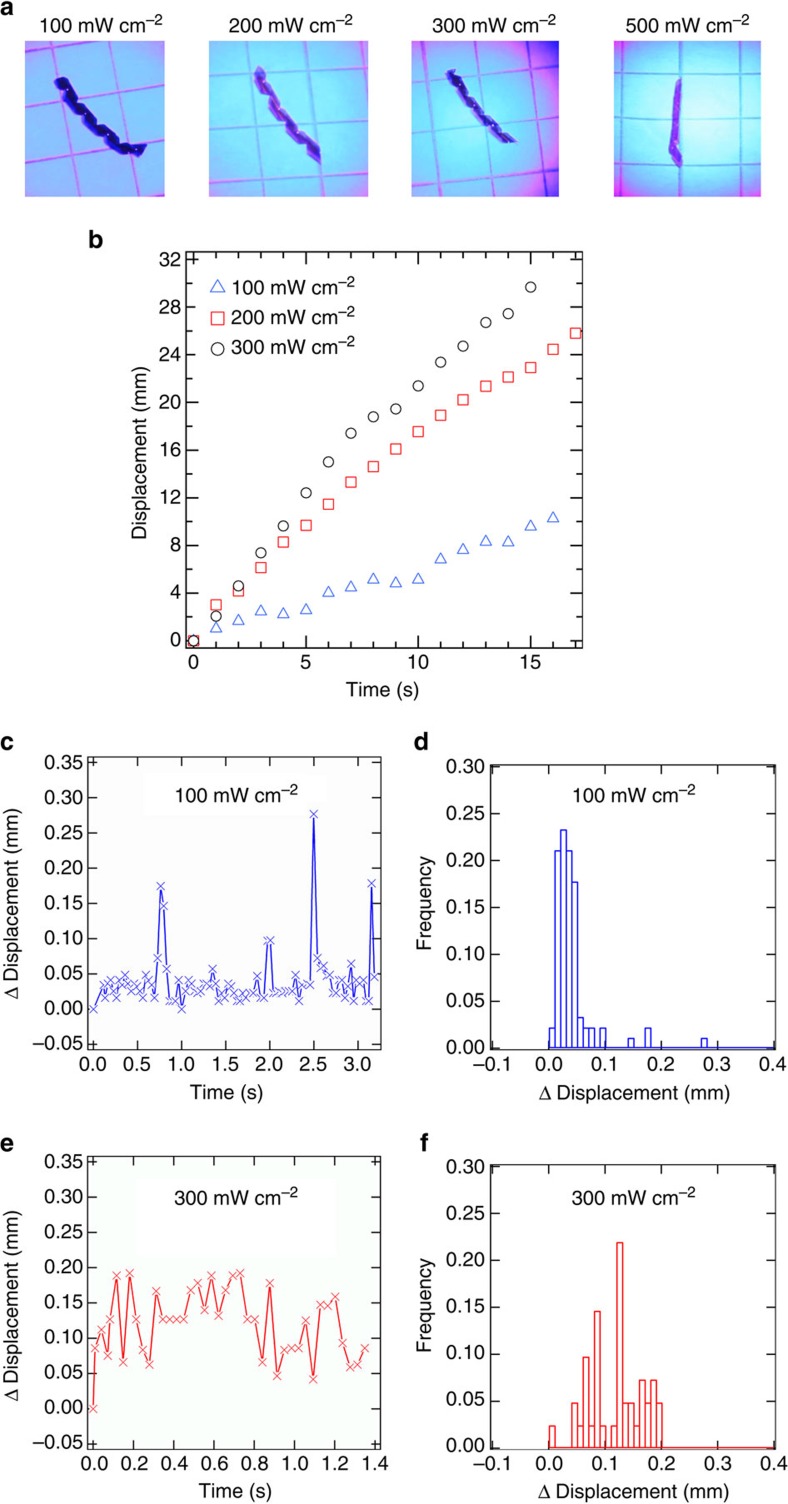
Contribution of light intensity. (**a**) Representative images of azo-LCN strips subjected to light intensities ranging from 100 to 500 mW cm^−2^. (**b**) Displacement of the strips (15° to −75° TN azo-LCNs) when subjected to 100, 200 and 300 mW cm^−2^ of light intensity. The relative displacement of the photomotile azo-LCN strips is recorded with 30 ms time interval for light intensity of 100 mW cm^−2^ (**c**) and 300 mW cm^−2^ (**e**). A histogram of normalized frequency *versus* displacement depicts the regularity of the motion at 100 mW cm^−2^ (**d**) and 300 mW cm^−2^ (**f**). The azo-LCN samples were 15 μm thick.

Photomotility is observed in specific conditions, particularly at aspect ratios of length to width greater than 3 (Supplementary Fig. 1) and sample thicknesses of 5–15 μm (Supplementary Fig. 2, Supplementary Movie 3) in the irradiation conditions attainable in our experiments. In the case of the 3 μm film, the tightness of the spiralled shape is such that the film is essentially a closed cylinder. Here, the interaction between the coils prevents the accumulation of the photostrains in a manner that allowed for motility evident in Figs 1 and 2. When the coils interact, they produce reaction forces intrinsically in the regions of self-contact that oppose further accumulation of the twist. The tightly coiled spiral produces blocking stresses from mutual constraint of the coils and suppresses the photoinduced strains. The inability to generate torque in the tightly coiled state eliminates the underlying cause of motility. The motion or lack thereof of these films to irradiation is evident in Supplementary Movie 3. The intensity of irradiation strongly influences the speed of the motion (Fig. 3a) evident in a plot of displacement as a function of time (Fig. 3b) and visualized in Supplementary Movie 4. The rolling motion, relating to the variance, as well as the average displacement are influenced by intensity, as shown in Fig. 3c–f. As evident hereto, motility is possible as long as sufficient moments can be photoinduced to overcome the resistance to rolling. If the responsiveness of the material to light is insufficient to create enough impulse and resulting τ_ph_ is too small to overcome friction and rolling resistance, the sample will remain immobilized on the surface. An illustrative case is found from the observation of limited motility in the lower intensity case (100 mW cm^−2^) in Fig. 3c. Increasing the intensity of irradiation is found to accelerate the velocity of the sample. This is expected because the rate of accumulation of photostrains scales with the intensity of irradiation. However, increasing the intensity does not indefinitely accelerate the photomotility. At very high intensities ∼500 mW cm^−2^, the spirals become very tightly coiled. The interaction of the coils with decreasing pitch generates intrinsic blocking stresses to prevent the realization of this underlying mechanism. This is consistent with that observed in other cases where inter–coil interaction precludes the activation of photomotility.

### Photoisomerization and thermal effects

**Figure 4:**
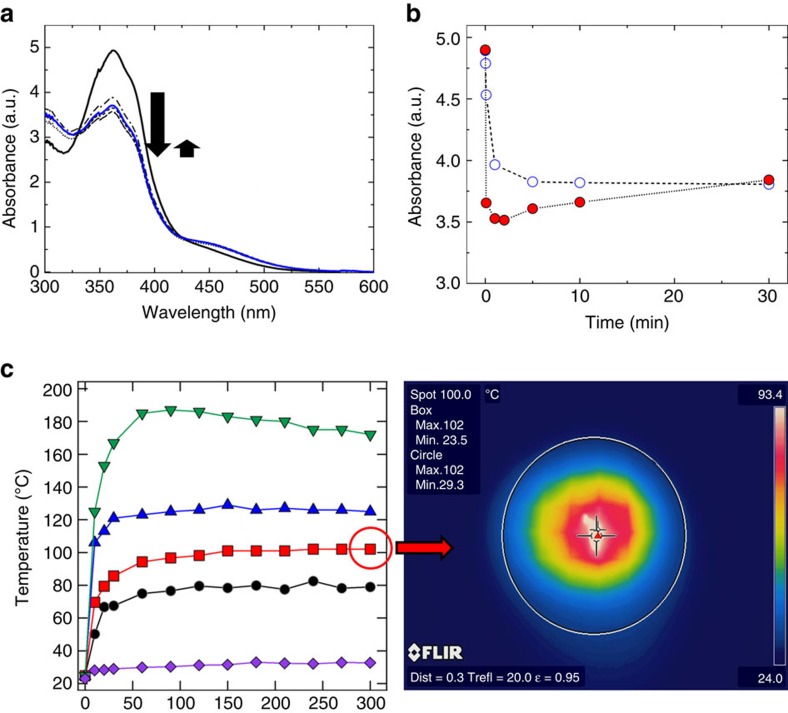
Photochemical and photothermal material responses. (**a**) UV-vis spectra of azo-LCN films with light (320–500 nm) irradiation at 200 mW cm^−2^ intensity. (**b**) Summary of ultraviolet–vis absorbance (at 365 nm) of azo-LCN films subjected to 30 mW cm^−2^ (Empty circle) and 200 mW cm^−2^ (Red filled circle) light intensities. (**c**) Thermal image and temperature evolution for azo-LCN films on exposure to the light intensities of 30 mW cm^−2^ (Purple color diamond), 100 mW cm^−2^ (Black filled circle), 200 mW cm^−2^ (Red filled square), 300 mW cm^−2^ (Blue color traingle) and 500 mW cm^−2^ (Green color inverted traingle).

In our experiments, the photomotility is ‘perpetual' in that we have not observed the films motion to stop under continuous irradiation as the material reaches and holds a photostationary state concentration enabled by the use of broad spectrum light. The photostationary state concentration of the azo-LCN material can be examined with absorption spectroscopy. As evident in Fig. 4a, the film initially exhibits a strong π–π* absorbance at 365 nm. After irradiation with the broadband light source at 200 mW cm^−2^, the π–π* absorbance peak rapidly decreases (indicated by the large downward pointing arrow in Fig. 4a) and the n–π* absorbance peak associated with the *cis* azobenzene isomer appears (smaller, upward pointing arrow). However, close examination of Fig. 4a, which is summarized in Fig. 4b shows that with continuous exposure the π–π* absorbance of the azo-LCN decreases but rises after sustained exposure. For comparison, the change in absorbance at 365 nm when an azo-LCN film of identical composition is irradiated with 30 mW cm^−2^ the film simply decays to a minimum photostationary state value. The inevitable question posed of this and prior examinations of photomechanical effects in polymers is how much heat is generated through absorption of the chromophores? To answer this question, we imaged the azo-LCN films with a thermal (FLIR) camera to a range of exposure intensities from 30 to 500 mW cm^−2^. As a control, the exposure of the substrate (grid paper) was also measured (absent of the azo-LCN sample). Evident in Fig. 4c, irradiation of both the films as well as substrate (Supplementary Fig. S3), with the broadband source can induce a rise in temperature to as much as 180 °C. In the conditions of Figs 1, 2, 3, exposure to 200 mW cm^−2^ increases the temperature of the films to 100 °C and increases the temperature of the substrate to 80 °C. Thus, the increase in absorption observed in Fig. 4a,b is likely attributed to heating, which is known to shift the equilibrium photostationary state concentration of *cis* isomers. The photomotility observed in Figs 1, 2, 3 and Supplementary Information is attributed to both the photochemical conformational change with contribution from absorptive heating. Subjecting the materials to a convective thermal gradient simply generated an immobile coiled ribbon. It should be noted that due to the large crosslink density of the azo-LCN materials examined here, the materials do not undergo a nematic-isotropic phase transition and remain in the twisted nematic orientation. While it is not possible to obtain a snap-shot in time of the differences between the section of the spiral in the optical path *versus* that which is occluded, Fig. 4 nonetheless shows how the *cis-trans* back-reaction is accelerated due to thermal effects, even in the presence of irradiation. Thus, when a hitherto irradiated part of the spiral becomes occluded and is subjected to the thermal effects, relaxation of the photostrains would inevitably occur. This further assists and sustains a regenerative mechanism to enable persistence of the underlying mechanism and allows for the ‘perpetual' photomotility in our samples.

### Photoinduced climbing

**Figure 5:**
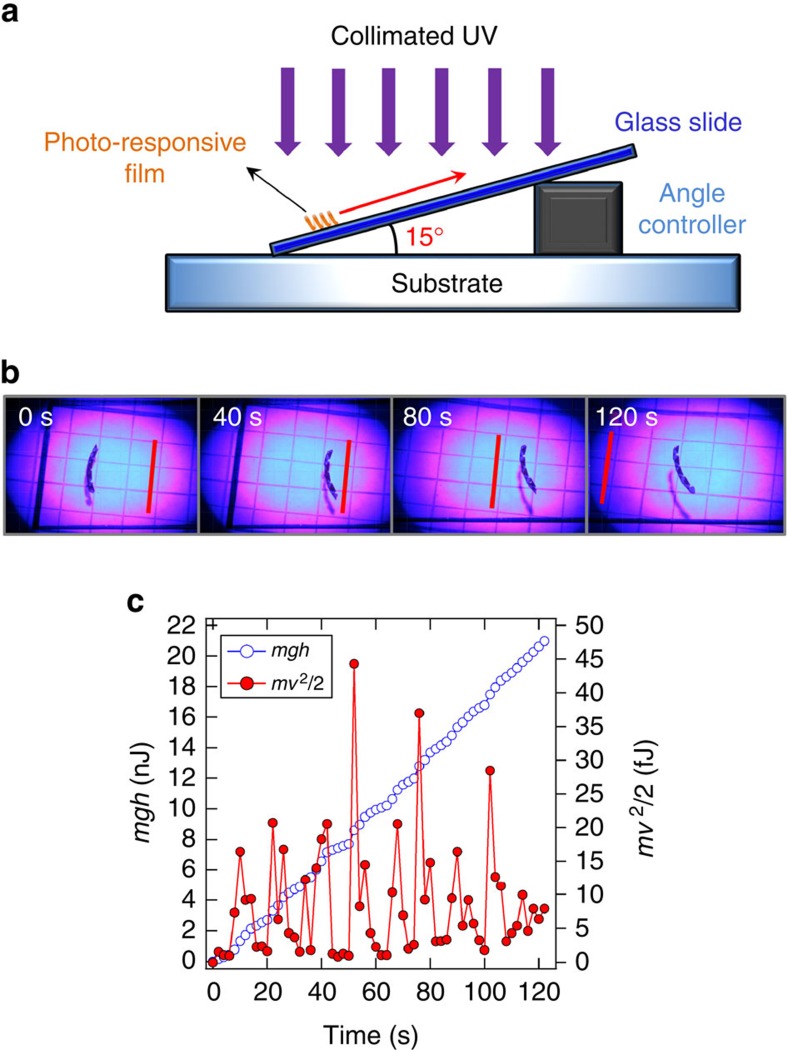
Defying gravity. (**a**) An illustration of climbing setup with a 15° incline. (**b**) Photomotility of the azo-LCN film on an incline is demonstrated at 40 s time interval. Red line is presented as a reference. (**c**) The work accomplished by the film is deconvoluted into potential (Empty circle) and kinetic (Red filled circle) contributions. The azo-LCN samples were 15 μm thick.

As illustrated in Fig. 5a, the sample was placed on a piece of glass that was set at a 15° incline. Evident in the images in Fig. 5b, on irradiation with 200 mW cm^−2^ the sample rolls up the incline at an average speed of 0.2 mm s^−1^. The red line inserted in the images of Fig. 5b is a common reference point, which illustrates the film rolling from left to right as it climbs the incline. Similar to the motion on flat surfaces, the film rolls intermittently from rest to displacement. The velocity measured in 30 ms intervals from the image sequences is used to calculate the kinetic energy in Fig. 5c. From the image analysis, the displacement (*h*) is measured in 30 ms intervals with respect to the horizontal to calculate the gravitational potential energy accumulated by the sample (Fig. 5c). The work performed against gravity in addition to the motility is *mgh*, where *m* is the mass of the sample, *g* is the acceleration due to gravity and *h* is the height. The change in *h* is measured in discrete time intervals of 30 ms. This illustrates the ability to extract finite mechanical work in addition to merely overcoming resistance and generating motility.

## Discussion

In summary, we demonstrate that on irradiation with broadband ultraviolet–visible light (320–500 nm) photoresponsive polymeric materials prepared here from azobenzene-functionalized liquid crystalline polymer networks spontaneously form spiralled structures that subsequently translate large distances on continuous irradiation. The locomotion of these materials is thus a direct conversion of the input light energy. The requisite conditions necessary to observe the photoinduced motion (photomotility) are described and depend on sample thickness, aspect ratio and light intensity. The directionality and rate of photomotility is dependent on the orientation of the liquid crystalline director to the principal axes of the sample as well as the light intensity. Photomotility may have potential advantages in miniaturized devices in that the remote and wireless power source for the mechanical response does not subject the functional devices to a weight penalty.

## Methods

### Materials

Photoresponsive azobenzene moiety, 4,4′-bis(6-(acryoloxy)hexyloxy)azobenzene (‘2-azo', BEAM Co.) was mixed with reactive liquid crystal monomer, 4-(3-acryloyloxypropyloxy)-benzoic acid 2-methyl-1,4-phenylene ester (‘RM257', Merck), photoinitiator, Irgacure 784 (‘I-784', Ciba) and chiral dopant (‘R1011', Merck) by a vortex mixer at a weight per cent of 20 wt%, 78.4 wt%, 1.5 wt% and 0.1 wt%, respectively. The mixture was heated to 120 °C for homogeneous mixing in an isotropic state and drawn into alignment layer (‘Elvamide', Dupont)-coated rubbed glass cells having twisted nematic geometry (90° alignment difference between top and bottom). The cell thickness was varied using inorganic spacers ranging from 3 to 50 μm. The molten mixture is subsequently cooled to 75 °C for nematic states and photopolymerized by 60 mW cm^−2^ of 532 nm laser light for 60 min. The liquid crystalline polymer film was harvested from the glass cell and cut with different film geometry and twisted nematic offset angles.

### Light source

Photomotility of the polymer film was initiated with a broad spectrum ultraviolet–visible irradiation (320–500 nm) to simultaneously generate *trans* and *cis* azobenzene for perpetual photomechanical motions. Light irradiation was generated by a Mercury bulb with a majority of the output in the ultraviolet at 365 nm as well as visible light emission at 410 nm (purple) and 442 nm (blue). The spectral output incident on the materials is cutoff at 500 nm with a filter (Supplementary Fig. 4).

### Imaging

A standard CCD camera was used to image the motion. Grid paper was utilized as a substrate to track displacement of the film. For uniform ultraviolet intensity over the entire polymer film, a collimator was placed on the exit of the fibre optic and centre region of the irradiated area was used for photoactuation. When polymer film escaped from the irradiated area due to the directional photomotility, the substrate was moved to relocate the film in the centre of the irradiated area for larger time-scale monitoring. However, relative displacement and histogram analysis was conducted only with the data when both the substrate and the beam were in a static mode. Frame by frame analysis of the motion was undertaken to produce the data presented in Figs 1, 2, 3 and 5. These data are representative of multiple trials.

### Light intensity

The full-spectrum light intensity was varied from 30 mW cm^−2^ to 500 mW cm^−2^. The intensity was measured using a Thorlabs power metre console. Collimated light was irradiated from top and the power metre was placed at the bottom with the identical distance between the light source and the sample. To confirm the uniformity of the light intensity, several different spots were tested.

### Temperature measurements

The photothermal effects from irradiation was monitored by forward looking infrared (FLIR) camera. The peak temperature value was recorded. The time-resolved temperature evolution on ultraviolet exposure was measured only with grid paper as a control and then was compared with the temperature with presence of light absorbing polymer film. The effect of light irradiation on photoisomerization of azobenzene was studied by temporal monitoring of ultraviolet–vis absorption spectra captured by a Cary 5000 spectrometer.

### Data availability

All data are available on request from the authors.

## Additional information

**How to cite this article:** Wie, J. J. *et al*. Photomotility of polymers. *Nat. Commun.* **7,** 13260 doi: 10.1038/ncomms13260 (2016).

**Publisher's note:** Springer Nature remains neutral with regard to jurisdictional claims in published maps and institutional affiliations.

## Supplementary Material

Supplementary Information Supplementary Figures 1-4

Supplementary Movie 1 Photomotility of azo-LCN film. Azo-LCN film aligned +15° to −75°, with dimensions 15 mm x 1.25 mm x 15 μm. Sample irradiated with 200 mW cm^−2^ of broadband UV light.

Supplementary Movie 2 Effect of twisted nematic offset angle. Azo-LCN films cut at angles ranging from 0°/-90°, 15°/-75°, 30°/-60°, 45°/-45°, 60°/-30°, 75°/-15°. All samples 15 mm x 1.25 x 15 μm in dimension, irradiated with 200 mW cm^−2^ of broadband UV light.

Supplementary Movie 3 Effect of film thickness. Azo-LCN films aligned 30°/-60° with dimensions 15 mm x 1.25 x 15 μm. Sample thickness varied from 3, 5, 15, 30, and 50 μm.

Supplementary Movie 4 Effect of UV intensity. Azo-LCN films aligned 30°/-60° with dimensions 15 mm x 1.25 mm x 15 μm. UV intensity ranging from 100, 200, 300, and 500 mW cm^−2^.
