# Supplementary material for: Photomotility of polymers
Source: Nat Commun. 2016 Nov 10;7:13260. doi: 10.1038/ncomms13260 (PMC5109552; doi:10.1038/ncomms13260)
Supplement: Supplementary Information — Supplementary Figures 1-4 [file ncomms13260-s1.pdf]

Supplementary Information

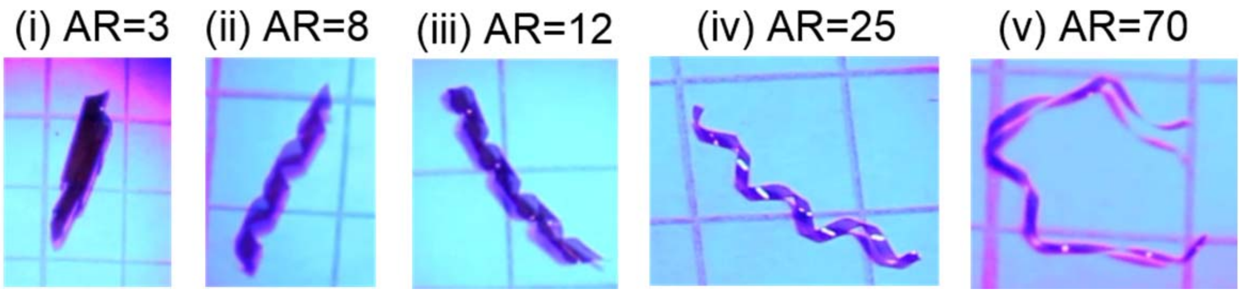

Supplementary Figure 1. Effect of film aspect ratio. Representative images illustrating the effect of aspect ratio (AR) on helical shapes and photomotility. Film width is changed to vary AR value with fixed film length (15 mm) and thickness (15 μm). Liquid crystalline alignment of +30° to -60° twisted nematic and UV intensity of 200 mW/cm^2 were utilized for all five samples. All films demonstrated photomotility except the film with AR=3.

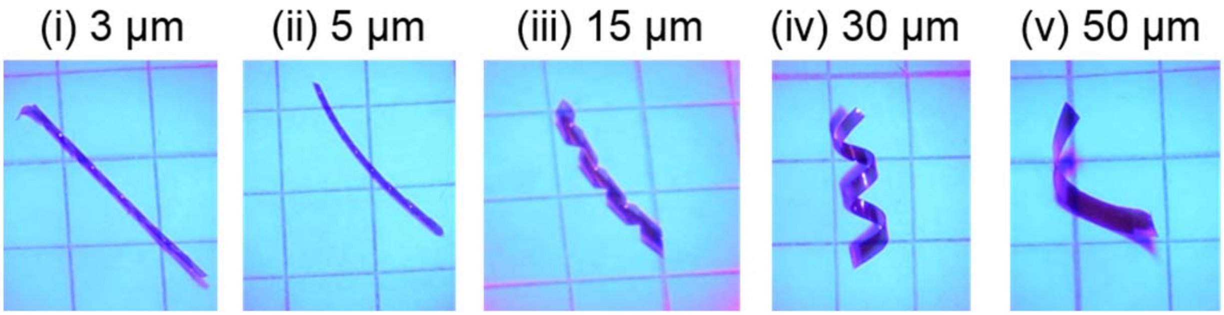

Supplementary Figure 2. Effect of film thickness. Representative images illustrating the effect of film thickness on helical shapes and photomotility. Film length and width were fixed as 15 mm and 1.25 mm, respectively. Liquid crystalline alignment of +30° to -60° twisted nematic and UV intensity of 200 mW/cm^2 were utilized for all five samples. Only 5 μm and 15 μm helical coils demonstrated photomotility.

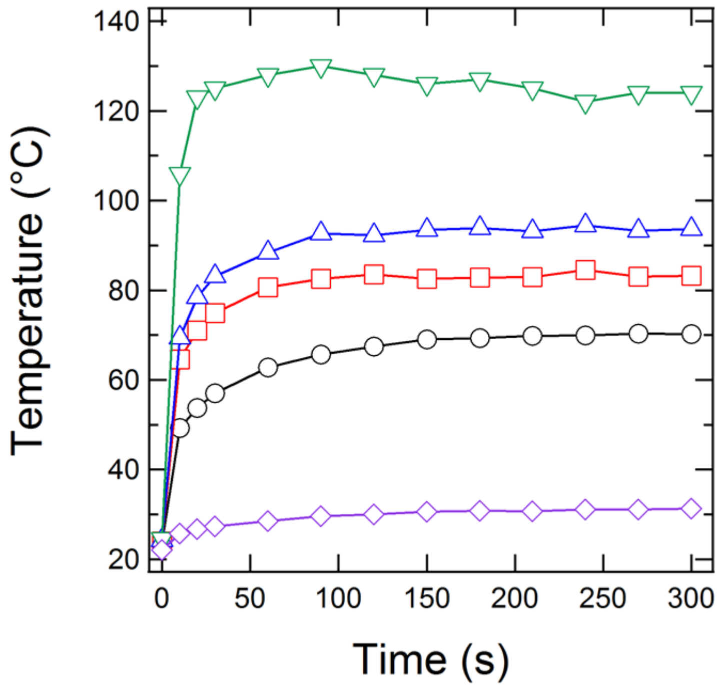

Supplementary Figure 3. Photothermal effect of UV on substrates. Time-resolved monitoring of substrate temperature without azo-LCN film by FLIR upon exposure to 30 mW/cm^{-2} (◊), 100 mW/ cm^{-2} (○), 200 mW/ cm^{-2} (□), 300 mW/ cm^{-2} (Δ), and 500 mW/ cm^{-2} (▽) of UV intensity.

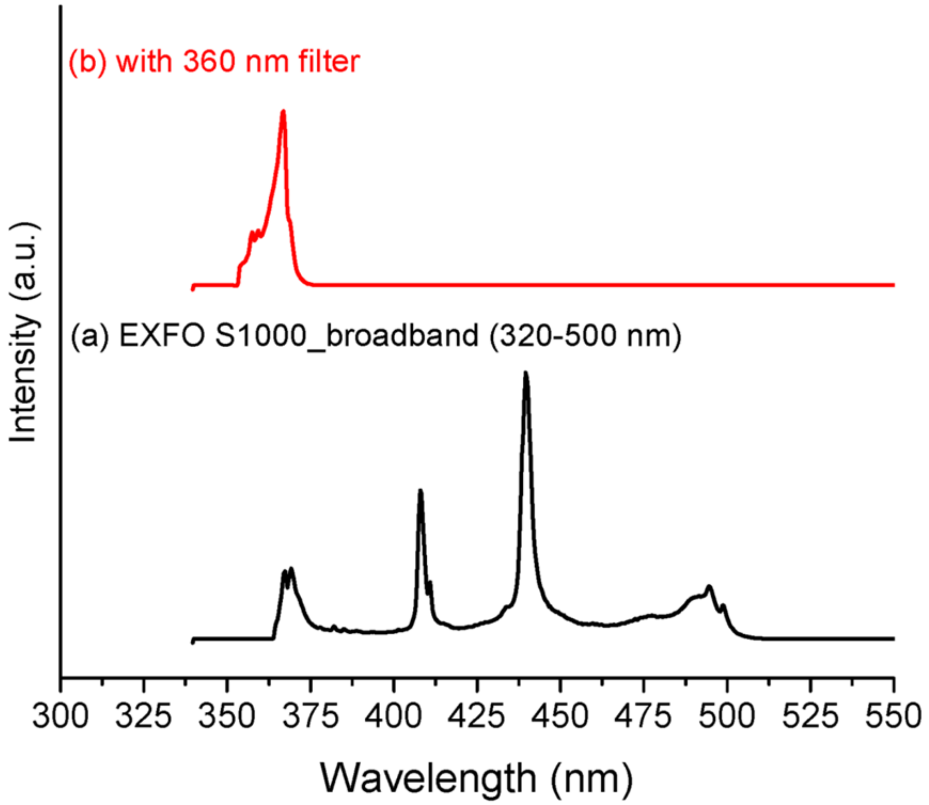

Supplementary Figure 4. Lamp emission. (a) The spectral emission of the mercury lamp employed here using a 320-500 nm filter. (b) Also shown, the spectral emission of the lamp when a 360 nm filter is inserted.
